# Rastislav Pjontek, Florian Scheibe, Julia Tabatabai (Hrsg.): Heidelberger Standarduntersuchung 

**DOI:** 10.3205/zma000955

**Published:** 2015-05-13

**Authors:** Florian Recker

**Affiliations:** 1 Rheinische-Friedrich-Wilhelms-Universität Bonn, Universitätsklinikum Bonn, Bonn, Deutschland

## Recesion

“For one mistake made for not knowing, ten mistakes are made for not looking.” (J.A. Lindsay).

“Looking” represents a cornerstone of the medical profession. Sir William Osler called it one of the “principles of practice” and added that “the whole art of medicine lies in observation” [1]. The introduction of percussion by Auenbrugger in 1761 and the invention of the stethoscope by Laënnec in 1819 helped doctors of the nineteenth century diagnose anatomical and pathological physiological conditions. These diagnostic advances led to a departure from the existing Bader practices.

At a time when ever more new invasive and non-invasive diagnostic procedures are available, the importance of the physical exam should be emphasized in clinical practice. Whoever manages to “gain more from their hands can be more relaxed and confident in everyday medical practice.” It is precisely the physical examination which forms the basis of all medical practice. It avoids long, harrowing journeys through the medical system and steers toward the appropriate path for a correct diagnosis.

Furthermore, the physical examination is an important medical ritual to strengthen the doctor-patient relationship and delivers additional salutary effects, whereas a poorly conducted examination can destroy this relationship. Studies suggest that both the environment and the quality of the physical examination may be connected to neurological changes in the patient [2].

Within this context, it is very pleasing to see that the *Heidelberger Standarduntersuchung* (Heidelberg Medical School, HeiCuMed) dedicates itself to this important topic, focusing primarily on the practical acquisition of these essential techniques. There is often a large discrepancy between theoretical knowledge and practical skills, particularly at the commencement of medical studies. The authors of this work – medical students and physicians themselves – provide an excellent overview of clinical examination methods for all disciplines divided over three sections.

The first section introduces the reader to basic clinical examination, for which the appropriate situation and physician conduct are also addressed. In addition to important fundamental techniques, anamnesis is also discussed. The book begins with a complete description of the examination process. Appropriate images illustrate the various steps of the process. A colour-coded legend provides references to the pitfalls, pathologies, scores and tips that are clinically relevant at each step.

The second section of the book is devoted to examination techniques in various clinical disciplines, ranging from paediatric exams to ENT techniques and post-mortems. These chapters are supplemented by impressive pathological photos and sketches, offering complete training that includes Swan Neck Deformity (p. 212) and the differential diagnosis of aphasia (p. 264).

The third and final part of the book includes an anatomical index linking the anatomical regions, the corresponding examinations, and various clinically important scores and scales, such as the APGAR score, Tanner stages, and geriatric assessment.

The authors consciously chose to obtain a Creative Commons license for their work, so that the content may be used freely in the classroom. This work is especially recommended for medical students as a perfect compendium and workbook, since it gives thorough interdisciplinary instructions for performing physical examinations. It guides the reader through all the main investigative steps and illustrates pathologies that may appear in the particular examination findings. All of this is complemented by access to an online portal with instructional videos.

The *Heidelberger Standarduntersuchung* is a perfect reference work for use by both students and practitioners of medicine.

## Competing interests

The author declares that he has no competing interests.
